# Latent class analysis–derived prediction model of shoulder function after arthroscopic repair for rotator cuff tear: a large retrospective cohort study

**DOI:** 10.3389/fsurg.2026.1876475

**Published:** 2026-09-03

**Authors:** Tingting Ma, Yun Wu, Lujian Liang, Yumeng Tang, Yang Li, Rui Yin, Xia Ming, Liyan Bian

**Affiliations:** 1 Department of Sports Medicine and Joint Surgery, Nanjing First Hospital, Nanjing Medical University, Nanjing, Jiangsu, China; 2 Department of Orthopedics, Nanjing First Hospital, Nanjing Medical University, Nanjing, Jiangsu, China

**Keywords:** arthroscopic rotator cuff repair, latent class analysis, multiple logistic regression, retear, shoulder function

## Abstract

**Objectives:**

Considering the potential probabilities of shoulder function following arthroscopic rotator cuff repair (ARCR) was crucial for clinical decision-making and postoperative managements. This study aimed to identify distinct patterns of postoperative shoulder function and their prognostic factors.

**Methods:**

Records of adult patients who underwent ARCR for the treatment of rotator cuff tear were reviewed from the electronic medical records. Constant–Murley scores were utilized to measure shoulder function at baseline and 3- and 12-month follow-ups. A latent class analysis was utilized to identify distinct classes of Constant–Murley trajectories. A multiple logistic regression analysis was conducted to examine risk factors.

**Results:**

A total of 553 patients were included in the study. Changes in postoperative shoulder function were best categorized into three latent classes: high function (Class 1, 65.7%), low function (Class 2, 24.1%), and high retear risk (Class 3, 10.2%, accounting for 71.7% of all retears). Patients in Class 1 experienced significantly greater improvements in shoulder function compared with those in both Class 2 and Class 3, achieving the highest Constant–Murley scores over 12 months following surgery. Using Class 1 as the reference, a regression analysis was performed, which identified risk factors for Class 2, including having at least two full-thickness tears, longer symptom duration, undergoing acromioplasty combined with capsulotomy, undergoing acromioplasty combined with acromioclavicular resection, lower nutritional risk index scores, having two or more elevated inflammatory cytokines, and using weak opioids for more than one postoperative month (all *p* < 0.05). For Class 3, risk factors included a massive tear, a global fatty degeneration index between 1 and 2 or >2, an intraoperative repair tension of 8 Ibf or more, and having at least two elevated inflammatory cytokines (all *p* < 0.05). The area under the receiver operating characteristic curves was 0.903 and 0.931 for the two models.

**Conclusions:**

Patients exhibited heterogeneous patterns of shoulder function following ARCR, each associated with different risk factors. These clinically distinct, trajectory-specific risk profiles provided actionable evidence to implement targeted, risk-stratified postoperative management protocols early after surgery. These findings emphasized the importance of individualized assessments and managements in the clinical setting, to reduce unfavorable outcomes and maximize postoperative shoulder function recovery for patients undergoing ARCR.

## Introduction

The rotator cuff consists of four muscles and their tendons, namely, the supraspinatus, infraspinatus, teres minor, and subscapularis, which plays a critical role in the dynamic stabilization of the shoulder (1). Rotator cuff tear is the most common tendon injury in adults, affecting approximately 30% of individuals over 60 years of age and 62% of those over 80 years of age (2). Chronic injuries typically arise from the degenerative changes of the rotator cuff, such as muscle atrophy, fibrosis, and fatty infiltration due to long-term overuse, which may occur with or without impingement. This condition progresses to tendinopathy, followed by partial-thickness damage, and eventually develops into a full-thickness tear if left untreated (3). Patients with rotator cuff tear usually experience shoulder pain that limits the flexion and internal and external rotation movements, leading to sleep disturbance, stress, and impairment in daily activities (4, 5). Arthroscopic rotator cuff repair (ARCR) is widely preferred because it is a minimally invasive surgical approach that preserves native anatomy, restores shoulder function, and improves quality of life (6). Despite its benefits, a subgroup of patients still suffer from postoperative shoulder pain, stiffness, and retears, which negatively impact the speed of patients’ recovery (7). Two recent reviews have summarized patient-specific factors that influence postoperative shoulder function; nevertheless, conventional regression models often rely on isolated outcome endpoints to predict functional performance (8, 9). In contrast, a latent class analysis (LCA) based on trajectory classification addresses this issue by grouping patients according to their sequential, repeated functional measurements over time following ARCR.

We hypothesized that distinct, clinically heterogeneous latent trajectories of shoulder function recovery exist following ARCR, with each trajectory associated with a unique set of independent prognostic risk factors. This study aimed to monitor and assess shoulder function using the clinician-reported Constant–Murley score over a 12-month follow-up period, and to investigate potential differences in postoperative recovery trajectories through the LCA. To facilitate early detection and intervention, predictive factors for improvements in shoulder function after ARCR were explored using a regression analysis based on the identified latent classes.

## Methods

### Study design and participant

The retrospective observational study using prospectively collected data was conducted between 1 January 2021 and 31 March 2025 at a large affiliated hospital of a comprehensive university in China. Ethical approval was granted by the Institutional Ethics Committee of Nanjing First Hospital, Nanjing Medical University (NFH-2026Y-031) in accordance with the principles of the Declaration of Helsinki. Results were reported following the Strengthening the Reporting of Observational Studies in Epidemiology (SRTOBE) guidelines for observational study (10). Written informed consent was obtained from all patients.

The medical records of patients admitted for the treatment of rotator cuff tear were reviewed from the electronic medical records (EMRs). The inclusion criteria were as follows: (1) meeting the diagnostic code for rotator cuff tear according to the International Classification of Diseases, 10th revision (ICD-10) (11); (2) confirmation by preoperative magnetic resonance imaging (MRI); (3) symptom duration of at least 3 months; (4) failure to respond to conservative treatments; (5) undergoing primary ARCR; and (6) age ≥18 years. Patients were excluded if they had other shoulder joint tears such as Bankart lesions and traumatic cuff injuries, shoulder infections, a history of shoulder surgery, convention to open surgery due to intraoperative exploration, severe physical illnesses, severe mental disorders, severe hearing problems, analgesic abuse, pregnancy or lactation, and incomplete medical data.

### Outcomes measurement

The patients’ demographic and clinical characteristics were retrieved from the EMRs. The Chinese version of the Constant–Murley questionnaire was utilized to measure shoulder function at baseline and 3 and 12 months following ARCR. It consisted of four subscales: two objective domains of pain (up to 15 points) and activities of daily living (up to 20 points), as well as two subjective domains of range of motion (up to 40 points) and strength (up to 25 points). The total score, ranging from 0 to 100, was calculated by summing all the item scores, with higher scores indicating better shoulder function (12). Fatty infiltration in cuff tears such as supraspinatus, infraspinatus, teres minor, and subscapularis was measured using the T1-weighted scapular Y view on MRI and categorized into five grades: Grade 0 (no fat), Grade 1 (some fatty streaks), Grade 2 (more muscle than fat), Grade 3 (equal amounts of fat and muscle), and Grade 4 (more fat than muscle). The global fatty degeneration index was determined by calculating the mean grade across all rotator cuff muscles according to the modified Goutallier classification, accurately reflecting the overall degenerative severity of the whole rotator cuff muscles (13). Shoulder stiffness was defined as a restriction in both active and passive mobility with global mobility＜50% and/or external rotation＜45° (14). Intraoperative repair tension was measured using a 12-Ibf tensimeter after the tendon was pulled to the planned attachment site. Surgeon and patient satisfaction with the surgery were assessed on a five-piont Likert scale ranging from 0 (very unsatisfactory) to 4 (very satisfactory) within 24 h following surgery. Serum levels of inflammatory cytokines such as interleukin-6 (IL-6), interleukin-8 (IL-8), interleukin-1β (IL-1β), and tumor necrosis factor-α (TNF-α) were tested at baseline and the 1-month routine follow-up after surgery. The nutritional risk index (NRI) score was calculated at 1 month postoperatively using the following formula: [1.519 × albumin (g/L)]＋41.7 × (present weight/ideal body weight) (15).

### Sample size calculation

PASS software, version 22 (NCSS, LLC., Kaysville, UT, USA) was used for sample size calculation. The minimal clinically important difference threshold of the Constant–Murley scores in patients undergoing ARCR was determined to be 6.7 points [95% confidence interval (CI): 4.5–8.9] based on the linear regression for satisfaction at 12 months postoperatively (16). The standard deviation (SD) was estimated to range from 5 to 10 based on recent studies (17, 18). The present study aimed for a sample size large enough to account for LCA with 2–6 classes and to detect a minimum detectable difference of 6.7 points among these classes. To achieve a 90% power with a two-sided significance level of 0.05, a total of 524 patients were required. After adjusting for a potential 20% data loss, the sample size was set at 656 cases.

### Statistical analysis

A statistical analysis was performed using SPSS software, version 22.0 (SPSS Inc., Chicago, IL, USA). A *p*-value of <0.05 was considered statistically significant. LCA was performed using Mplus 8.3 software to identify subgroups among patients undergoing ARCR based on their postoperative shoulder function. Different polynomial types such as linear, quadratic, and cubic were tested. Subsequently, the number of classes was tested to determine the best-fitting model. The best-fitting solution was selected based on several criteria, including the following: (1) the smaller values of the Akaike information criterion (AIC), Bayesian information criteria (BIC), and adjusted Bayesian information criteria (aBIC) indicated a better fit; (2) an entropy value greater than 0.8 suggested a classification accuracy of approximately 90%; (3) the Vuong–Lo–Mendell–Rubin likelihood-ratio test (LMR LRT) and bootstrapped likelihood ratio test with *p*-values <0.05 indicated statistically significant improvements in fit with additional categories.

A Kolmogorov–Smirnov *Z* test was used to assess data normality. Normally distributed continuous data and categorical data were described as mean ± SD and percentages, respectively. Differences among the latent classes were evaluated using one-way analysis of variance (ANOVA) with the *post hoc* Bonferroni test (adjusted *p* = 0.017) and Chi-square test. The variance inflation factor (VIF) was utilized to assess multicollinearity among variables, with a VIF of 10 or higher indicating a serious collinearity issue. Multiple binary logistic regression using the likelihood ratio test was employed to identify the independent factors affecting the postoperative shoulder function. Receiver operating characteristic (ROC) curves were generated to estimate the diagnostic accuracy of each predictive model. Patients with missing data were excluded from the analysis.

## Results

**Figure 1:**
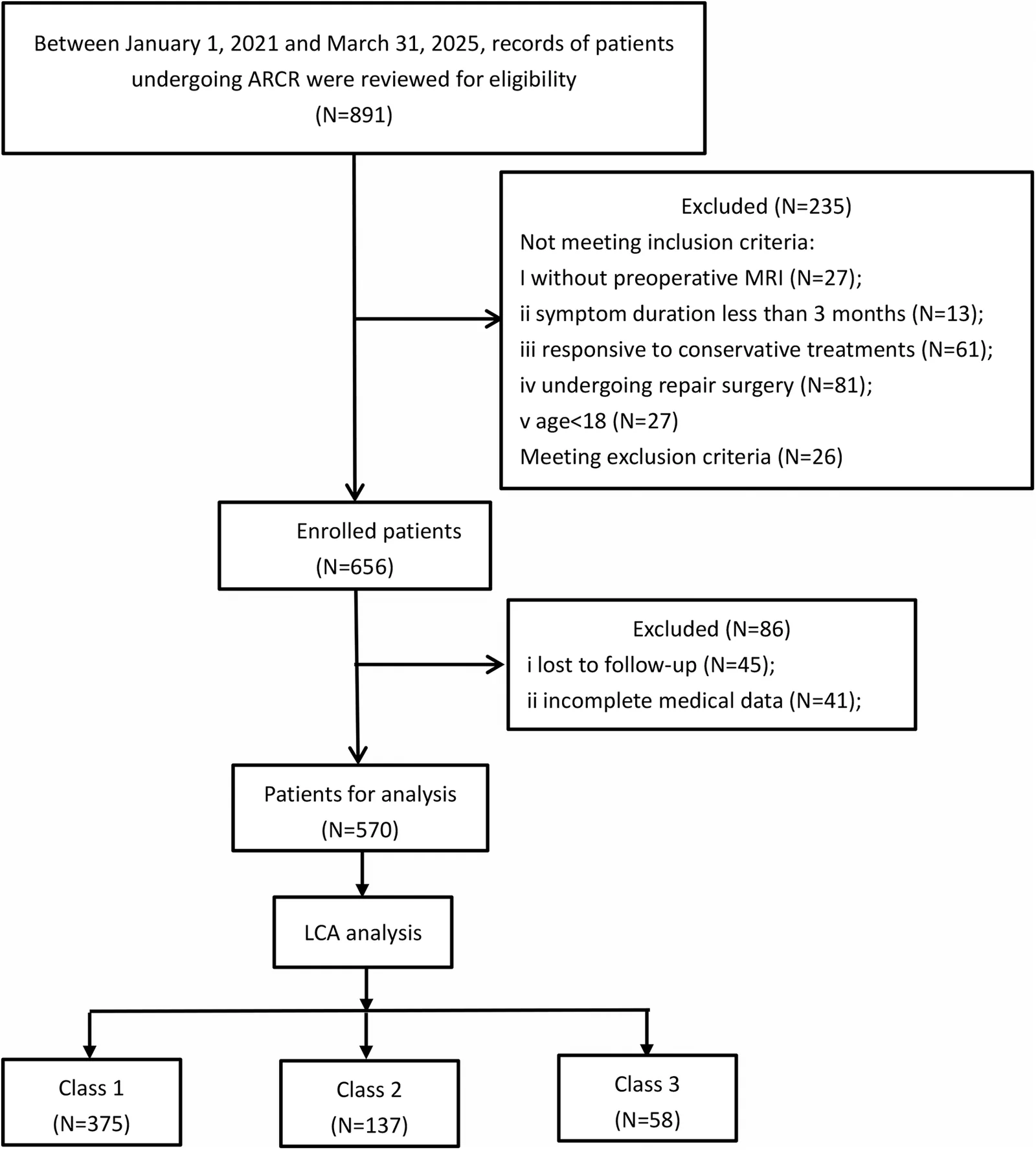
A flowchart of the study cohort. ARCR, arthroscopic rotator cuff repair; MRI, magnetic resonance imaging; LCA, latent class analysis.

Figure 1 displays a flowchart of the entire study cohort. A total of 656 patients were enrolled. After the preliminary review of all electronic medical records, 13.1% of cases were excluded because of incomplete medical data that could not be supplemented or verified, leaving 570 individuals included in the final analysis cohort, which was consistent with the sample size requirements calculated in advance.

The three-class model exhibited the highest entropy value up to 0.988. This value was superior to the entropy of the two-class (0.944), four-class (0.879), five-class (0.881), and six-class (0.861) models, indicating an excellent model fit and strong classification accuracy. The LMR test *p*-value for the three-class model was less than 0.001, demonstrating that it delivered a statistically significant improvement in model fit compared with the two-class model. Although the AIC, BIC, and aBIC values declined as the number of model categories increased, the three-class model maintained a low set of information criterion values that were better than those of the two-class model. The smallest class in the three-class model accounted for 10.2% of the total cohort, which meets the widely accepted minimum sample proportion threshold, ensuring sufficient statistical power to carry out stable multiple logistic regression for subsequent risk factor identification. Therefore, the model with three potential categories was selected as the optimal model after comprehensively evaluating all performance metrics listed in Table 1.

**Table 1:** Model fit for LCA.

Latent classes	Parameters	AIC	BIC	Adjusted BIC	Entropy	Smallest class (%)	Log-likelihood	LMR *p*-value
2 classes	10	11,755.822	11,799.279	11,767.533	0.944	33.2%	−6,054.316	<0.001
3 classes	14	11,454.961	11,515.800	11,471.356	0.988	10.2%	−5,867.911	<0.001
4 classes	18	11,269.610	11,347.832	11,290.690	0.879	10.2%	−5,713.480	<0.001
5 classes	22	11204.453	11,300.057	11,230.217	0.881	10.2%	−5,616.805	<0.001
6 classes	26	11,167.131	11,280.118	11,197.580	0.861	9.3%	−5,580.226	<0.001

LCA, latent class analysis; AIC, Akaike information criterion; BIC, Bayesian information criteria.

**Figure 2:**
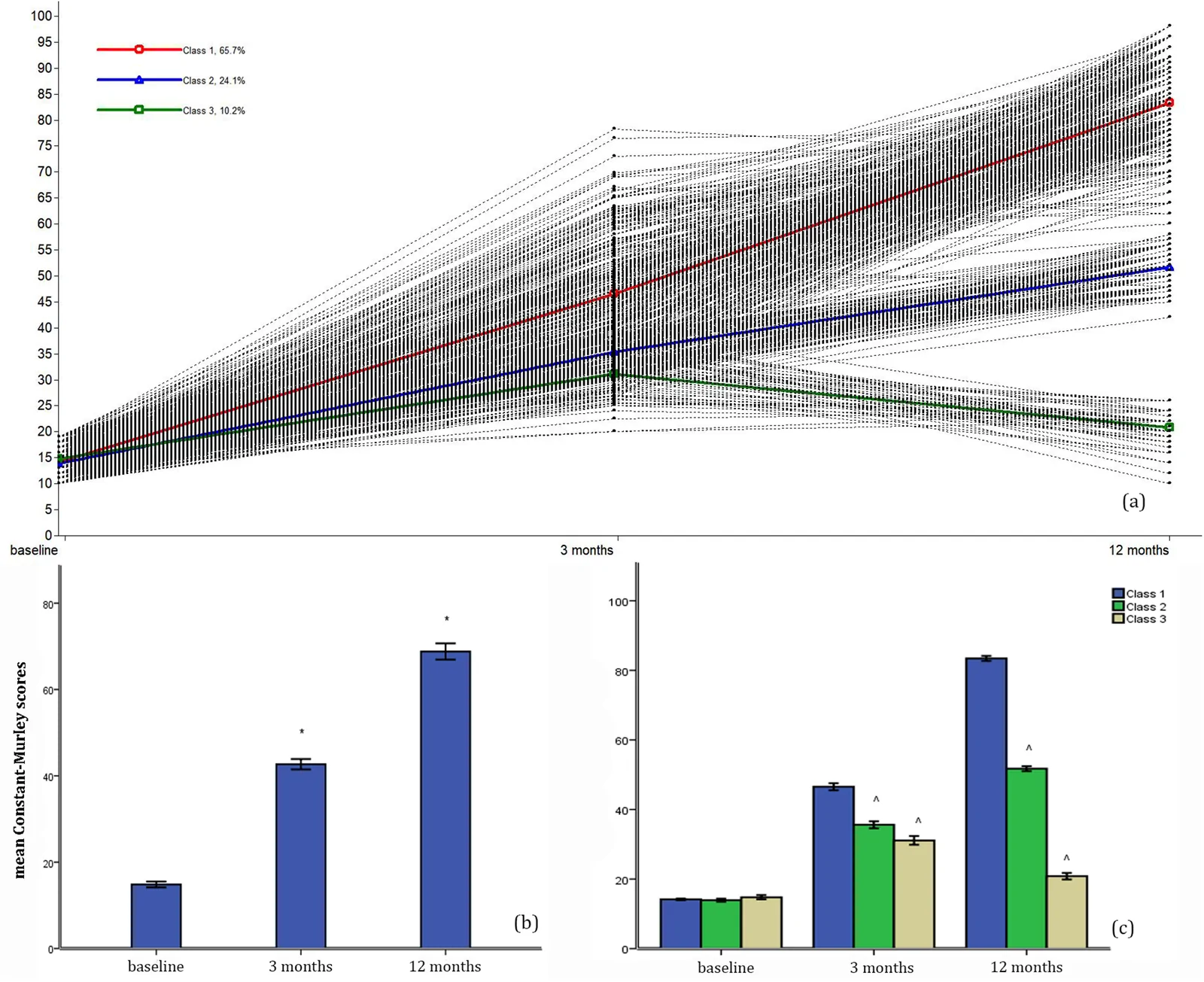
Constant–Murley scores over 12 months after ARCR. **(a)** LCA identified three different trajectories based on the observed individual trajectories from 570 patients; **(b)** Mean Constant–Murley scores for all patients during a 12-month follow-up; **(c)** Mean Constant–Murley for three classes across all time points over 12 months. ARCR, arthroscopic rotator cuff repair; LCA, latent class analysis. *p < 0.05 compared with the baseline value and ^*p* < 0.05 compared with the Class 1 cohort.

Figure 2a shows the estimated trajectories for the three latent classes. In this model, 65.7% of patients who experienced significantly high improvements in shoulder function over 12 months following surgery were categorized into Class 1 (high shoulder function). Meanwhile, 24.1% of individuals with low improvements in shoulder function were assigned to Class 2 (low shoulder function), and the remaining 10.2% of cases who showed a temporary, minimal improvement in shoulder function were classified into Class 3 (high risk of retear), with a retear rate of 74.1%, accounting for 71.7% of the total retear rate. As illustrated in Figure 2b, the mean Constant–Murley scores significantly increased from a baseline value of 14.16 ± 2.51–42.30 ± 10.55 at 3 months and to 69.32 ± 22.03 at 12 months after surgery (both *p* < 0.001). However, an ANOVA analysis revealed significant differences among the three latent classes at both 3 months (*F* = 132.241, *p* < 0.001) and 12 months (*F* = 3147.561, *p* < 0.001) postsurgery. Furthermore, Class 2 and Class 3 showed significantly lower Constant–Murley scores compared with Class 1 at both 3-month [mean difference (MD) of −10.92 for Class 2 and −15.41 for Class 3, both adjusted Bonferroni *p* < 0.001] and 12-month (MD of −31.67 for Class 2 and −62.583 for Class 3, both adjusted Bonferroni *p* < 0.001) follow-ups (Figure 2c).

As shown in Table 2, significant differences were observed among the three latent classes in terms of symptom duration before surgery, tear size, tear severity, Goutallier fatty infiltration grades, global fatty degeneration index, concomitant procedures, anchors used, postoperative inflammatory cytokines, NRI scores, postoperative analgesic use, and postoperative exercise (all *p* < 0.05). All variables with a *p*-value below 0.20 across the three latent classes in Table 2 were enrolled into the pool of candidate variables. After multicollinearity diagnostics were conducted, all test variables showed a VIF of less than 10. According to multiple binary logistic regression, the following were identified as independent risk factors for low shoulder function, taking the latent Class 1 as the reference cohort: having at least two full tears [odds ratio (OR) = 1.206], longer symptom duration before surgery (OR = 1.248), undergoing both acromioplasty and capsulotomy (OR = 4.805), undergoing both acromioplasty and acromioclavicular resection (OR = 6.774), lower postoperative NRI scores (OR = 3.266), having ≥2 elevated inflammatory cytokines after surgery (OR = 8.022), and using weak opioids for more than 1 month following surgery (OR = 3.579) (all log-rank test *p* < 0.05). In addition, when categorizing Class 1 as the favorable trajectory and Class 3 as the unfavorable trajectory, the regression results indicated that a massive tear (OR = 3.233), a global fatty degeneration index of ＞1 and ≤2 (OR = 5.740)/＞2 (OR = 7.429), an intraoperative repair tension ≥8 Ibf (OR = 4.791), and having ≥2 elevated inflammatory cytokines after surgery (OR = 2.660) were associated with a higher risk of retear after ARCR (Table 3). Other variables could not exert an independent predictive effect after the covariates retained in the final model were adjusted, because they failed to reach the *p* < 0.05 significance threshold in the multivariable likelihood ratio test.

**Table 2:** Demographic and clinical characteristics of patients undergoing ARCR at baseline.

Variables	LCA			*F*/*χ*^2^ value	*p-*	All patients (*n* = 570)
	Class 1 (*n* = 375)	Class 2 (*n* = 137)	Class 3 (*n* = 58)			
Age, years (mean ± SD)	62.55 ± 10.62	63.02 ± 9.58	61.83 ± 11.21	0.273	0.761	62.06 ± 10.85
Sex, *n* (%)				0.071	0.965	
Female	201 (53.6%)	73 (53.3%)	30 (51.7%)			304 (53.3%)
Male	174 (46.4%)	64 (46.7%)	28 (48.3%)			266 (46.7%)
Race, *n* (%)				0.436	0.804	
Han Chinese	271 (72.3%)	95 (74.8%)	41 (70.7%)			407 (72.7%)
Minority	104 (27.7%)	42 (25.2%)	17 (29.3%)			153 (27.3%)
BMI, kg/m^2^ (mean ± SD)	23.91 ± 3.73	24.64 ± 3.82	23.59 ± 4.06	2.348	0.097	22.62 ± 3.65
Affected side, *n* (%)				0.224	0.894	
Left	143 (38.1%)	53 (38.7%)	24 (41.4%)			220 (38.6%)
Right	232 (61.9%)	84 (61.3%)	34 (58.6%)			350 (61.4%)
Education status at least high school, *n* (%)	144 (38.4%)	51 (37.2%)	22 (27.9%)	0.059	0.971	217 (38.1%)
Symptom duration before surgery, m (mean ± SD)	12.86 ± 9.20	15.15 ± 7.23	13.49 ± 8.93	3.444	0.033	13.42 ± 9.89
Tear type, *n* (%)				0.565	0.967	
Acute traumatic tear	47 (12.5%)	14 (10.2%)	7 (12.1%)			68 (11.9%)
Sport tear	67 (17.9%)	26 (19.0%)	11 (19.0%)			104 (18.2%)
Chronic degenerative tear	261 (69.6%)	97 (70.8%)	40 (69.0%)			398 (69.8%)
De Orio and Cofield classification for tear size, *n* (%)				13.482	0.009	
Medium (1–3 cm)	45 (12.0%)	20 (14.6%)	6 (10.3%)			71 (12.5%)
Large (3–5 cm)	272 (72.5%)	89 (65.0%)	32 (55.2%)			393 (68.9%)
Massive (＞5 cm)	58 (15.5%)	28 (20.4%)	20 (34.5%)			106 (18.6%)
Tear severity, *n* (%)				11.557	0.021	
Only partial tear	46 (12.3%)	16 (11.7%)	5 (8.6%)			67 (11.8%)
Only one full tear	255 (68.0%)	75 (54.7%)	38 (65.5%)			368 (64.6%)
At least two full tears	74 (19.7%)	46 (33.6%)	15 (25.9%)			135 (23.7%)
Retraction grade, *n* (%)				0.683	0.953	
Grade I (near the footprint)	106 (28.3%)	41 (29.9%)	19 (32.8%)			166 (29.1%)
Grade II (at humeral head midpoint)	189 (50.4%)	66 (48.2%)	28 (48.3%)			283 (49.6%)
Grade III (at glenoid level)	80 (21.3%)	30 (21.9%)	11 (19.0%)			121 (21.2%)
Goutallier fatty infiltration grades on MRI, (mean ± SD)						
Supraspinatus	1.47 ± 0.96	1.56 ± 0.98	2.25 ± 0.92	16.561	<0.001	1.89 ± 0.96
Infraspinatus/teres minor	1.81 ± 0.83	1.88 ± 0.86	2.43 ± 0.91	13.514	<0.001	1.97 ± 0.94
Subscapularis	1.49 ± 0.74	1.57 ± 0.85	2.19 ± 1.31	17.420	<0.001	1.92 ± 0.97
Global fatty degeneration index, *n* (%)				33.097	<0.001	
≤0.25	76 (20.3%)	26 (19.0%)	10 (17.2%)			112 (19.6%)
＞0.25 and ≤1	195 (52.0%)	70 (51.1%)	23 (39.7%)			288 (50.5%)
＞1 and ≤2	104 (27.7%)	39 (28.5%)	20 (34.5%)			163 (28.6%)
＞2	0 (0%)	2 (1.5%)	5 (8.6%)			7 (1.2%)
Critical shoulder angel, degrees (mean ± SD)	35.43 ± 3.78	35.91 ± 4.02	35.67 ± 3.96	0.799	0.450	35.73 ± 3.86
Acromiohumeral interval on X-ray, (mean ± SD)	5.84 ± 2.27	5.63 ± 2.33	5.42 ± 2.35	1.077	0.341	5.67 ± 2.30
Range of motion, degrees (mean ± SD)						
Abduction	125.43 ± 40.53	124.58 ± 41.68	128.97 ± 41.64	0.242	0.785	126.03 ± 40.94
Flexion	138.24 ± 36.78	137.15 ± 38.22	135.39 ± 37.27	0.166	0.847	137.78 ± 36.42
External rotation	58.25 ± 17.51	56.29 ± 18.44	57.11 ± 16.34	0.652	0.521	57.36 ± 18.96
Internal rotation	46.72 ± 24.69	44.55 ± 23.38	43.31 ± 25.78	0.741	0.477	44.10 ± 26.09
Preoperative shoulder stiffness, *n* (%)	85 (22.7%)	49 (35.8%)	16 (26.3%)	8.934	0.011	150 (26.3%)
Preoperative weak opioids use, *n* (%)	69 (18.4%)	27 (19.7%)	13 (22.4%)	0.563	0.755	109 (19.1%)
Preoperative steroid injection, *n* (%)	58 (15.6%)	22 (16.1%)	11 (19.0%)	0.413	0.814	91 (16.1%)
Preoperative Douleur Neuropathique-4 score, (mean ± SD)	1.89 ± 1.13	2.95 ± 1.48	4.36 ± 1.60	111.318	0.660	2.58 ± 1.99
Preoperative osteoporosis in shoulder joint, *n* (%)	45 (12.0%)	17 (12.4%)	8 (13.8%)	0.153	0.927	70 (12.3%)
Common physical comorbidities, *n* (%)						
Hyperlipemia	78 (23.0%)	30 (24.5%)	12 (22.4%)	0.208	0.901	132 (23.9%)
Hypertension	58 (15.5%)	19 (14.3%)	10 (17.1%)	0.451	0.798	88 (15.9%)
Diabetes	48 (12.8%)	23 (13.0%)	11 (22.1%)	0.036	0.982	71 (12.8%)
Other	53 (14.2%)	17 (12.1%)	8 (10.3%)	0.683	0.711	69 (12.5%)
Surgical technique, *n* (%)				0.303	0.990	
Single row	63 (16.8%)	25 (18.2%)	11 (19.0%)			99 (17.4%)
Double row	168 (44.8%)	61 (44.5%)	26 (44.8%)			255 (44.7%)
Suture brigading	144 (38.4%)	51 (37.2%)	21 (36.2%)			216 (37.9%)
Concomitant procedure performed, *n* (%)						
Long head of biceps tenotomy/tenodesis	87 (23.2%)	37 (27.0%)	15 (25.9%)	0.865	0.649	139 (24.4%)
Acromioplasty	86 (22.9%)	48 (35.0%)	12 (20.7%)	8.536	0.014	146 (25.6%)
Acromioplasty and capsulotomy	32 (8.5%)	23 (16.8%)	6 (10.3%)	7.164	0.028	61 (10.7%)
Acromioplasty and acromioclavicular resection	26 (6.9%)	22 (16.1%)	5 (9.3%)	15.932	0.007	53 (9.3%)
Intraoperative increasing pressure irrigation, min (mean ± SD)	6.83 ± 3.72	6.58 ± 3.86	6.90 ± 4.16	0.251	0.778	6.68 ± 4.44
Intraoperative remifentanil consumption, µg (mean ± SD)	322.45 ± 165.51	319.49 ± 151.48	320.95 ± 144.46	0.017	0.983	321.94 ± 159.25
Bleeding volume, mL (mean ± SD)	53.98 ± 8.57	55.73 ± 8.02	54.20 ± 8.63	2.176	0.114	54.77 ± 8.56
Anchors used, *n* (%)				9.167	0.010	
＜5	313 (83.5%)	98 (71.5%)	45 (77.6%)			456 (80.0%)
≥5	62 (16.5%)	39 (28.5%)	13 (22.4%)			114 (20.0%)
Surgery time, min (mean ± SD)	81.75 ± 10.74	80.21 ± 12.30	83.52 ± 11.90	1.919	0.148	83.86 ± 11.19
Intraoperative acromion morphology, *n* (%)				0.143	0.998	
Flat	102 (27.2%)	38 (27.7%)	15 (25.9%)			155 (27.2%)
Curved	202 (53.9%)	73 (53.3%)	31 (53.4%)			306 (53.7%)
Hooked	71 (18.9%)	26 (19.0%)	12 (20.7%)			109 (19.1%)
Intraoperative repair tension, *n* (%)				14.226	0.007	
0–3 Ibf	148 (39.5%)	54 (39.4%)	13 (22.4%)			215 (37.7%)
4–7Ibf	163 (43.5%)	60 (43.8%)	24 (41.4%)			247 (43.3%)
≥8 Ibf	64 (17.0%)	23 (16.8%)	21 (36.2%)			108 (18.9%)
In-hospital analgesic requirement, (mean ± SD)						
Fentanyl in postoperative PCA, µg	5658.17 ± 1068.48	5862.67 ± 1248.21	5758.08 ± 1154.60	1.706	0.182	5690.13 ± 1048.62
Diclofenac consumption, mg	122.14 ± 18.54	124.29 ± 12.48	126.68 ± 15.67	2.221	0.109	124.50 ± 16.73
Surgeon satisfaction following surgery, (mean ± SD)	3.13 ± 0.99	3.36 ± 0.75	3.12 ± 0.92	1.399	0.248	4.16 ± 0.98
Patient satisfaction following surgery, (mean ± SD)	3.32 ± 0.94	3.42 ± 0.93	3.34 ± 0.95	0.211	0.810	4.34 ± 0.95
Arm strength at discharge (mean ± SD)	5.54 ± 1.87	5.59 ± 1.71	5.48 ± 1.88	0.479	0.620	5.45 ± 1.79
Hospital stays, days (mean ± SD)	7.73 ± 1.90	7.65 ± 1.28	7.49 ± 1.74	0.509	0.602	7.58 ± 1.65
Inflammatory cytokines at postoperative 1 month, pg/mL (mean ± SD)						
IL-6	3.49 ± 2.38	4.27 ± 2.84	5.28 ± 2.63	15.026	<0.001	4.39 ± 2.51
IL-8	8.26 ± 3.15	8.52 ± 4.73	9.37 ± 3.64	4.114	0.017	8.34 ± 4.01
IL-1β	2.36 ± 1.42	3.47 ± 1.72	4.38 ± 1.96	57.360	<0.001	3.68 ± 1.84
TNF-α	6.54 ± 2.14	8.39 ± 2.43	8.73 ± 2.32	49.526	<0.001	7.03 ± 2.82
NRI scores at postoperative 1 month, (mean ± SD)	109.36 ± 6.61	92.83 ± 6.55	93.38 ± 6.14	400.648	<0.001	95.39 ± 6.56
Analgesic use ＞1 month after surgery, *n* (%)				93.974	<0.001	
None	333 (88.8%)	98 (71.5%)	29 (50.0%)			460 (80.7%)
NSAID	35 (9.3%)	27 (19.7%)	10 (17.2%)			72 (12.6%)
Weak opioid	7 (1.9%)	12 (8.8%)	19 (32.8%)			38 (6.7%)
Postoperative exercise, *n* (%)				25.438	<0.001	
Adherence to prescription	280 (74.7%)	82 (59.9%)	40 (69.0%)			402 (69.0%)
Delayed mobility exercise	64 (17.1%)	41 (29.9%)	5 (8.6%)			110 (19.3%)
Early mobility exercise	31 (8.3%)	14 (10.2%)	13 (22.4%)			58 (10.2%)

ARCR, arthroscopic rotator cuff repair; BMI, body mass index; MRI, magnetic resonance imaging; PCA, patient-controlled analgesia; NRI, nutritional risk index; NSAID, non-steroidal anti-inflammatory drug; SD, standard deviation; Ibf, pound-force.

**Table 3:** Prediction of three latent classes of shoulder function after ARCR based on multiple logistic regression.

Variables		Regression coefficient			Adjusted hazard ratio			*p*
		*B*	SE	Waldχ^2^	Exp (B)	95% CI		
						Lower	Upper	
	Tear severity							
	Partial or one full tear							
	At least two full tears	0.187	0.047	15.633	1.206	1.099	1.323	<0.001
	Symptom duration before surgery	0.222	0.023	96.522	1.248	1.194	1.305	<0.001
	Concomitant procedure performed			11.849				0.008
	Without acromioplasty							
	Acromioplasty	0.726	0.438	2.750	2.067	0.876	4.875	0.097
	Acromioplasty and capsulotomy	1.570	0.536	8.571	4.805	1.680	13.743	0.003
	Acromioplasty and acromioclavicular resection	1.913	0.689	7.716	6.774	1.756	26.125	0.005
Class 2 vs. Class 1	NRI scores at postoperative 1 month							
	Normal							
	Lower than mean value	1.183	0.432	7.510	3.266	1.401	7.613	0.006
	Inflammatory cytokines at postoperative 1 month							
	Normal							
	At least two elevated cytokines	2.082	0.488	21.601	8.022	3.334	19.302	<0.001
	Analgesic use ＞1 month after surgery			8.379				0.039
	None							
	NSAID	1.084	1.571	1.918	2.956	0.136	6.238	0.166
	Weak opioid	1.257	0.503	6.426	3.579	1.335	9.592	0.011
	Constant	−27.126	3.088	77.180	0.001			<0.001
	De Orio and Cofield classification for tear size							
	Medium to large							
	Massive	1.170	0.354	10.900	3.233	1.609	6.456	0.001
	Global fatty degeneration index			11.849				0.008
	≤0.25							
	＞0.25 and ≤1	0.346	0.546	0.402	1.414	0.458	4.124	0.526
Class 3 vs. Class 1	＞1 and ≤2	1.747	0.713	6.009	5.740	1.419	13.208	0.014
	＞2	2.005	1.010	3.940	7.429	1.026	23.805	0.047
	Intraoperative repair tension							
	＜8 Ibf							
	≥8 Ibf	1.567	0.721	4.724	4.791	1.166	9.682	0.030
	Inflammatory cytokines at postoperative 1 month							
	Normal							
	At least two evaluated cytokines	0.978	0.355	7.607	2.660	1.327	5.332	<0.001
	Constant	−2.447	0.792	9.546	0.087			0.002

ARCR, arthroscopic rotator cuff repair; NRI, nutritional risk index; NSAID, non-steroidal anti-inflammatory drug; CI, confidence interval; Ibf, pound-force.

**Figure 3:**
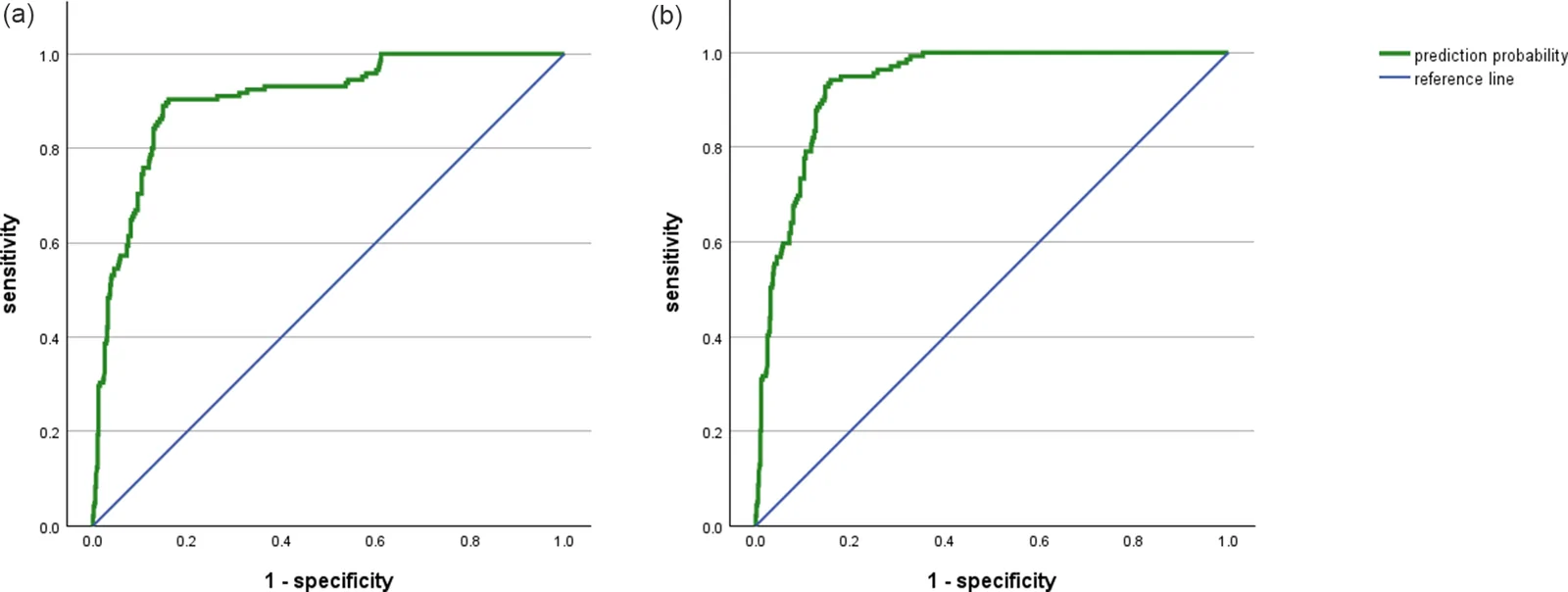
ROC curves of two multiple logistic regression models. **(a)** The model taking high postoperative shoulder function class as favorable and low function class as the reference achieved an AUC of 0.903 (95% CI: 0.876-0.930); **(b)** The model taking high postoperative shoulder function class as favorable and high risk of retear class as reference achieved an AUC of 0.931 (95% CI: 0.912-0.953). ROC, receiver operating characteristic; AUC,  area under curve; CI, confidence interval.

ROC curves were generated, and the area under the curve (AUC) was calculated as 0.903 [95% CI: 0.876–0.930] for the Class 2 vs. the Class 1 model and 0.931 (95% CI: 0.912–0.953) for the Class 3 vs. the Class 1 model. The Youden index, sensitivity, and specificity for the two models were 0.741, 89.7%, and 84.1% and 0.782, 93.5%, and 84.3%, respectively (Figure 3). The Hosmer–Lemeshow test yielded a *p*-value of 0.214 for the Class 2 vs. the Class 1 model and 0.187 for the Class 3 vs. the Class 1 model, indicating no significant discrepancy between the predicted risk probabilities and the actual observed event rates. The calibration-in-the-large scores for the two models were 0.002 and −0.001, respectively.

## Discussion

To the best of our knowledge, this study was the first to identify the distribution patterns of postoperative shoulder function over time in patients undergoing ARCR using LCA. Our findings showed three trajectory patterns with distinct characteristics: high function, low function, and high risk of retear, providing important insights into their associated risk factors.

A recent meta-analysis and systematic review have indicated that ARCR is an effective treatment option for patients who have failed conservative therapies, including those over 75 years of age (19). A more recent prospective cohort study reported that Constant–Murley scores increased from a baseline value of 16.3 ± 4.1–45.2 ± 8.1 at 3 months and 70.1 ± 6.9 at 12 months after ARCR for treating rotator cuff tear (20). Consistent with earlier findings, our results showed overall mean Constant–Murley scores of 42.30 at 3 months and 69.32 at 6 months postsurgery, respectively. Nevertheless, three latent classes of postoperative shoulder function were identified based on individual trajectories, indicating distinct probabilities of shoulder function improvements after surgery. These differences should be taken into account when making decisions in clinical settings.

According to a systematic review, patients who underwent ARCR within 3 months after symptom onset showed a 19-point greater improvement in Constant–Murley scores and were three times more likely to achieve acceptable shoulder pain levels, with a mean VAS score of 1.7 points (8). Stojanov et al. developed a model predicting patient-reported shoulder function after ARCR using medical data from 1,510 patients in a Swiss setting. Their findings revealed that patients with more than one fully torn tendon had a 1.83-fold increased risk of poor postoperative functional outcomes (21). In line with these findings, our results showed that a shorter symptom duration and the presence of two or more fully ruptured tendons were significantly associated with lower improvements in Constant–Murley scores. A recent study found that acromioplasty improved the Penn total score and Penn-function score after ARCR (22). However, unlike earlier research studies that showed no differences in shoulder function between patients who underwent ARCR with or without acromioplasty, the current study revealed that patients undergoing concomitant procedures such as acromioplasty combined with capsulotomy (OR = 4.805) and acromioplasty combined with acromioclavicular resection (OR = 6.774) were more likely to experience lower improvements in postoperative shoulder function compared with those in the reference Class 1 cohort (23). These results might be attributed to the prevalence of shoulder stiffness, a markedly reduced acromiohumeral interval or acromioclavicular joint arthritis accompanied by directed osteophytes, all indicating more complex conditions that were significantly associated with worse functional scores after ARCR (24, 25). Furthermore, patients whose NRI scores were below the mean value and who had at least two elevated inflammatory cytokines reported 3.266 times and 8.022 times higher risks, respectively, of exhibiting lower Constant–Murley scores. Recent evidence reported that better nutritional status, indicated by higher prognostic nutrition index scores, was identified as a significant independent protective factor against both muscle atrophy (OR = 0.822, 95% CI: 0.720–0.938, *p* = 0.004) and fatty infiltration (OR = 0.801, 95% CI: 0.700–0.918, *p* = 0.001), resulting in less severe rotator cuff muscle degeneration and better functional outcomes following ARCR (15). In addition, increased concentrations of IL-1β, IL-6, and TNF-αwere found in the joint fluid of patients with rotator cuff tears, which correlated with severe motion limitations and worse Constant functional scores (26). For patients with the abovementioned risk factors, additional shoulder mobilization, and strength sessions, nutritional support might be included, while the use of weak opioid should be restricted to prevent worsening functional impairment.

In the present study, 10.5% of patients experienced retears during a 12-month follow-up period, with 71.7% of these retears occurring in Class 3 patients. A recent retrospective study involving 262 patients who underwent ARCR demonstrated that a higher proportion of massive tears was observed before surgery in the retear cohort (15% vs. 3.1%, *p* = 0.044) (27). Furthermore, more severe fatty infiltration was considered as an independent risk factor for retear, with ORs ranging from 4.5 to 7.5 according to prior findings (27, 28). Our findings also indicated that patients with a preoperative massive tear, a fatty degeneration index between 1 and 2, or >2 had a 3.233-fold, 5.740-fold, and 7.429-fold higher risk, respectively, of experiencing a retear following ARCR. Kai-Lan Hsu et al. investigated the relationship between repair tension and radiologic outcomes and found that poor healing after ARCR occurred in 25.4% of patients, with a significant difference in repair tension (*p* = 0.002) (29). Sam-Guk Park et al. reported that the cutoff value for repair tension was 7.87 Ibf; tension exceeding this threshold was the strongest predictor of retear, with an AUC of 0.799, a sensitivity of 84.2%, and a specificity of 67.7% (30). Our findings supported this evidence, showing that compared with patients in Class 1 (the relevant reference cohort), those with intraoperative repair tension greater than 8 Ibf were 4.791 times more likely to develop a retear within 12 months postsurgery. In addition, patients who had at least two elevated inflammatory cytokines at 1 month postoperatively exhibited a 2.660-fold increased risk of experiencing a retear. This might be attributed to the mechanism whereby rotator cuff tears activated tenocytes and recruited immune cells to produce multiple inflammatory cytokines, exacerbating adverse changes in the tendon structure (31). Therefore, individuals who have a high risk of retear should be prescribed strict shoulder abduction brace protection, avoid heavy lifting and high-resistance shoulder activities, and undergo a routine MRI re-examination for at least 6 months after ARCR. Early anti-inflammatory treatments should be initiated at 1-month follow-up for patients exhibiting two or more elevated inflammatory cytokines.

There were several limitations in this study. First, dependence on retrospective data might introduce some undetected confounders and potential biases. Second, we did not check whether the patients excluded from the analysis differed meaningfully from those included in the final cohort. Third, the LCA method inherently depended on a specific statistical assumption of conditional independence of repeated outcome measurements, which might not completely capture all underlying data complexity. Fourth, the three-class model fitted well with our single-center cohort; it required external validation across different ethnic and clinical populations to confirm its reliability and generalizability. Fifth, some predictors included in our model were measured after surgery, which aimed to capture patients’ early responses and the dynamic changes during recovery process, helping to guide personalized postoperative rehabilitation strategies. However, because this model was not strictly a “preoperative clinical prediction model,” it could not be directly used to guide the decision-making process before surgery. Last, although the reported AUC values were promising, the absence of both internal and external validations posed a risk of overfitting, limiting the generalizability of our results. Therefore, our findings need to be confirmed by a well-designed randomized controlled trial in the future.

In conclusion, our findings revealed three distinct trajectories of shoulder function following ARCR. Using patients with high postoperative Constant–Murley scores as a reference, this study identified different risk factors associated with low shoulder function and a high risk of retear within 12 months after ARCR. Although these findings may assist surgeons in their decision-making and postoperative managements, it is important to note that external validation is required before these models can be recommended in clinical practice.

## Data Availability

The raw data supporting the conclusions of this article will be made available by the authors, without undue reservation.
